# Worldviews and Conflict Analysis

**DOI:** 10.1111/nejo.12411

**Published:** 2022-08-22

**Authors:** Mona Kanwal Sheikh

**Keywords:** conflict analysis, worldviews, worldview analysis, conflict resolution

## Abstract

This article lays out an argument for relocating worldview analysis from the margins of conflict analysis to its center. While we may understand worldviews as an integral part of most escalated conflicts—which may seem to be about something else as well (e.g., energy, borders, economic grievances)—worldviews conflict can also be described as a particular form of conflict. This duality is important to recognize for the further development of the field of conflict analysis. The article also lays out the relevance of worldview analysis to conflict analysis, and how it can enhance our understanding of escalatory conflict dynamics.

## Introduction

Debates about worldviews and their significance for understanding human behavior and collective identities are not new. Scholars from many academic disciplines have their own reasons for engaging with worldviews. As I have described in an earlier review of how different social science disciplines approach worldview analysis (Sheikh 2018, 2019),^1^ social psychologists are primarily interested in the ways human beings encounter the world; they “study the formation of collective attitudes and the way different communities relate to one another” (Sheikh 2019: 114). The goal of their analysis is to understand processes of othering, including strategies that communities develop to guard themselves when facing perceived threats from competing worldviews (Greenberg, Solomon, and Pyszczynski 1997; McGregor et al. 2001; Koltko‐Rivera 2004; Major et al. 2007; Goplen and Plant 2015).

Anthropologists seek to describe and compare certain ways of life (Sapir 1949; Redfield 1952; Hiebert 2008). Since worldview analysis in anthropology centers on an interest in, for example, ideas about the good life or how communities develop different customs and institutions, anthropologists focus less on the cognitive and perceptive dimensions of worldviews and more on the “affective, cultural and moral dimensions” (Hiebert 2008: 15).

Political scientists' interest in worldviews reflects a focus on how ideas and beliefs influence political decisions such as the use of force (Goldstein and Keohane 1993; Blyth 1997; Røislien 2007; Rowland and Theye 2008; Bottici and Challand 2010). Worldviews in the context of the social sciences may thus be understood either as impacting human action or as justifying certain actions (Sheikh 2018, 2019).

For the field of conflict analysis, it is important to understand the nexus between worldviews on one side and human affect and identity formation on the other, as well as the nexus between worldviews, sensemaking, and action, i.e., worldviews and their “outward effects.” The risk of portraying political opponents and perpetrators of violence as reflections of pure evil is that such an approach ignores the significance of worldviews in the acts of such individuals. Worldview analysis is thus all of this at once, including the “how‐question,” i.e., how conflict actors view the world in ways that allow them to carry out violent actions. Koltko‐Rivera (2004) contends that what makes worldviews intriguing objects of scientific inquiry is not whether they exist per say, but whether they are powerful in shaping affect, cognition, and behavior (23). The goal is, as explained in earlier work, to understand a framework for thinking about reality and acting appropriately within a perceived understanding of the world (Juergensmeyer and Sheikh 2013).

In this article, I first argue for the need to relocate worldview analysis from the margins to the center of conflict analysis. It is after all not only about “them,” those at the margins of international society, but equally about “us,” since having an outlook on the world is a universal condition. While worldviews might be understood as an integral part of most escalated conflicts that might seem to be about something else as well (energy, borders, economic grievances, etc.), worldview conflict can also be described as a particular form of conflict.

Secondly, in this article I move on to define a worldview, drawing on some earlier work on the concept developed by myself and Mark Juergensmeyer. However, in this article, such work is applied for the purpose of underpinning the argument that worldviews are something transhuman, something that we all possess. The final sections highlight the relevance of worldview analysis to conflict analysis, and what it can enhance by including it into our analyses when it comes to our understanding of escalatory conflict dynamics. These sections hence reflect on the relationship between worldviews and conflict behavior and relate worldview analysis to conflict theory.

## From the Margins to the Center of Conflict Thinking

Worldview analysis is relevant not only to understanding those at the fringes of society; it is equally important in understanding the mainstream. In my work on worldview analysis with Mark Juergensmeyer, we have applied it primarily as an approach to study religious zealots, though we have recognized the need to apply it to a broader range of actors, those who could be categorized as secular as well as religious (Sheikh and Juergensmeyer 2019). In the field of terrorism studies, there has been a tendency to approach and understand violence in connection to certain ideologies and ideas, including myths and religious doctrines (Rowland and Theye 2008; Edwards 2014). This sometimes has created the impression that religion explains violence. This tendency—as I will explain below—can be avoided through worldview analysis. Consideration of the mythic aspects of extremists' worldviews illuminates the narratives that underpin violent acts, including suicide bombings, massacres, and other actions carried out in the name of jihad or another cause or movement. Worldview analysis has until now proven particularly helpful for understanding those at the margins of society, those whose political motivations are rendered unintelligible in dominant public discourse. These include violent political activists, anti‐democracy activists, and suicide‐attackers. We can learn, for example, that in the mind of a militant jihadist, the act of beheading is not considered a brutal act of evil, but a purposeful act to defeat evil powers fighting Islam. And in the eyes of a suicide‐bomber, they are not perpetrating attacks on innocents or committing suicide but undertaking a self‐sacrifice that increases one's standing in the eyes of God. Understanding the rationales obviously does not justify such acts but it can sharpen our ability to think about solutions in ways that do not play into an escalatory conflict dynamic.

Worldview analysis is not, however, relevant only to understanding religious violence or those at the margins of society. Such analysis should be considered as a more foundational approach, one that helps us to also understand those in the mainstream or the violence associated with a secular vision of the world and secular values, such as freedom and liberty. Whether we are talking about the French Revolution, the Cold War, or the more recent War on Terror, all these events were expressions of large‐scale identities and visions of the world that (to some people) justified extraordinary measures and violence.

One may trace the worldview concept to many different thinkers but one obvious inspiration is Immanuel Kant (1790). In his work *Critique of Judgment*, Kant (1790) introduced the concept of *Weltanschauung* to describe the human ability to create order in a complex world that may be perceived in infinite ways. Thus, for Kant, a worldview is a comprehensive lens through which human beings see and experience the world and come to terms with it (Kant 1987).

As I have described in my review of the social science literature on worldview analysis (Sheikh 2019), this Kantian way of describing a worldview is easily paired with what psychologists call cognitive schemata—tools that allow human beings to reduce the complexity of experience in order to cope better with reality (Beck et al. 1979; Fiske and Linville 1980; Hastie 1981; Janoff‐Bulman 1989; DiMaggio 1997; Koltko‐Rivera 2004). The cognitive approaches have adopted Kant's understanding of worldviews as universally applicable and as reflections of the *human ability* to create order, meaning, and value in a chaotic world (Janoff‐Bulman 1989; Hiebert 2008; Johnson, Hill, and Cohen 2011).

The social constructivist understanding of the worldview concept reflects another perspective on why worldviews matter. From such perspective it is not so much the human mind that is interesting, but the social effect of narrating/constructing a worldview that dignifies communities/individuals, identifies adversaries, and links communities/individuals to a worldly or otherworldly purpose (Rowland and Theye 2008; Edwards 2014; cf. Røislien 2007). Rowland and Theye (2008) define worldviews as an “epistemic genre” that “explain[s] the person's place in the world, provide[s] a sense of reborn identity, identif[ies] a villain to be fought, and link[s] the individual to a transcendent purpose that can be achieved through violence” (56). This definition is in line with what Juergensmeyer and I have described as an epistemic worldview—a concept that combines Michel Foucault's work on the episteme, which is “the structure of knowledge behind an understanding of how reality works, and Pierre Bourdieu's notion of habitus, the social location of shared understandings about the world and how it should work” (Juergensmeyer and Sheikh 2013; Sheikh 2019: 112).

The social constructivist way of understanding a worldview adds an additional layer to the definition of worldview by pointing out that *ethics* is one of the elements that organize worldviews (Goldstein and Keohane 1993; Koltko‐Rivera 2004). This means that worldviews are interpretations of how the world is (ontological and descriptive) and that they also contain visions about how it ought to be (normative and prescriptive). One could pair this with the division suggested by Goldstein and Keohane (1993), who on the one hand talk about *principled beliefs* referring to ideas of good and bad, right and wrong, just and unjust; and on the other discuss *causal beliefs* that are prescriptions for attaining the good, right, and just (Goldstein and Koehene 1993; see also Bottici and Challand 2006; Rowland and Theye 2008).

Thus, worldviews also are connected to emotions and, I argue, cannot be conceptualized as an intellectual, ideological, or doctrinal category only. Particular narratives and framings are linked to particular moral codes and ethics (e.g., a particular ethics of just war). Therefore, worldviews should not be approached as a purely cognitive phenomenon without consideration of the emotional dimensions of mobilization. This tendency to approach worldviews as primarily a cognitive phenomenon also has been dominant in the field of cultural sociology, where attempts to approach the individual through frames, narratives, and discourses have generally been silent about emotions (Goodwin et al. 2001). The absence of attention to emotions reinforces a tendency in political science to focus on leaders and their way of framing events or manipulating the masses, leaving out the all‐important issue of the social dynamics of the masses and their receptiveness to particular framings.

Worldview analysis attempts to have a broad approach to different outlooks on the world, but also to understand the reality of a particular worldview: its social, ethical, political, and spiritual aspects and how they come together into a coherent whole (Juergensmeyer and Sheikh 2013). This analysis is, furthermore, concerned with worldviews from the perspectives of those who articulate them (in contrast to objectivist or Marxist‐inspired approaches that might claim a privileged insight into why people act the way they do) and is therefore close to the methodologies dominant within the fields of ethnography and anthropology. Anthropologist Robert Redfield (1952) stated that “world view refers to the way the world looks [to] people looking out” (30). In worldview analysis, narratives are taken seriously not only as superficial rhetoric, but as claims that can shape the social reality of human beings as well as their justifications for acting in certain ways. Contrary to Marxist analyses of human speech and behavior as falling back on the structural conditions of the individual or its group, worldview analysis considers speech and behavior in light of the particular life situation of the one who expresses them.

There are a different set of discursive practices that have developed around the application of worldviews that is observant to the reasoning and narrative of the study object as well as the social setting that conditions it. In contrast to how ideology often has been studied in the field of political science, the guiding idea in worldview analysis is not to verify or falsify the ideas people hold or provide better explanations than the subjects themselves, but to bring these ideas to the fore. What matters are the concepts they use, the categories they invoke, the imagery they paint, and the systems of representation that are part of their stories about the world or the conflict in which they are involved.

In worldview analysis, social reality is created in a dynamic relationship with articulations and expressions about reality. Hence, social reality—including different truths about the world—cannot be understood or accessed separately as an external fact, but is in itself generated by speech acts; it is not external to the “mind” or to “speech” (Harré and Gillet 1994: 22). Thus, the analysis of worldviews as proposed by Juergensmeyer and Sheikh (2013) does not focus on questions such as: Do people really mean what they say? Are they using their words strategically to hide their real beliefs or motives for actions? To what extent are they even aware of their real reasons for acting in certain ways? The validity of claims is not determined by whether utterances are true or convincing, but whether they seem to be important for the persons making them.

## Worldviews and Behavior—Avoiding the Traps

Worldview analysis can take different directions but there are three important traps to avoid when conducting it. The first is the assumption that worldviews and civilizations or other large‐scale identities overlap. The second is the proposition that worldviews determine actions and hence precede actions. And the third is an essentialist and static concept of worldviews that is detached both from those who hold the views and from their interactions with the social world (Sheikh 2019, 2021). I already have described the last trap in the discussion above, but the first two need further clarification.

One of the main criticisms of, for example, Samuel P. Huntington's (1993, 1996) proposition about world conflict has been that it rests on the idea that religions and civilizations are coherent, and by extension, can explain what causes conflicts. It is not rare for worldviews to overlap with other social boundaries (ethnic, tribal, or religious) and there can be concentric circles of social realities that coalesce with particular worldviews (Juergensmeyer and Sheikh 2013). However, as observers, we cannot assume a lack of variation or a high degree of overlap between different social identities, as this risks intensifying or reinforcing the imageries behind worldview conflicts, which must remain open for examination to avoid simplistic and generalizing usages of “a Muslim worldview,” “a Christian worldview,” and so on.

The other point of caution concerns causality. As observers—scholars or practitioners—we have access to the narratives of our subjects. To avoid assumptions about causality, which are methodologically problematic to measure precisely since we do not have direct access to the inner workings of human minds, one approach is to examine the conditions under which we can find a stronger relationship between worldviews and acts of violence or conflict. Some worldviews are characterized by low levels of ambiguity and high levels of simplicity, and one could hypothesize that they are more likely to enable or justify extreme action. When actors have strong ethical beliefs, a clear sense of identity, unambiguous justificatory narratives, and simple visions of ways to achieve their objectives, the motivation to act uncompromisingly to achieve their ends increases greatly. Narratives in which the world is seen through cosmic war images—and in which adversaries are identified as absolute enemies and one's own group is perceived as part of a heroic vanguard—are more likely to lead to stronger mobilizations in violent or confrontational directions. Huntington's description of civilizational clash can be useful if instead of understanding it as an essentialist diagnosis of world conflicts, it is applied as a lens that can display how some conflict actors perceive and organize the world. Imagined worlds—regardless of whether they reflect truth—can be powerful mobilizers, as they draw on a wide reservoir of ethical perceptions, collective memories, and emotions.

Rather than embrace a simple and linear cause‐effect relationship in which x leads to y (Sheikh 2019), an alternative option is to relate x to y as an enabling factor. Worldviews thus play a role in human behavior by “leading it onto certain action tracks, while obscuring other tracks” (Goldstein and Keohane 1993: 12). Bourdieu's work on the concept of classification struggles (1994) has shown how human struggles to classify the social world denote struggles over the dominant “principles of vision and di‐vision” and that social communities are formed through these processes (Bourdieu 1980: 483; 1990: 138).

Bourdieu is not focused on future wars between cultures, but on how cultural conflict is played out in a political context, i.e., the ways in which powerful actors represent their opponents and position themselves, and how their truth claims are projected to create dominance over others (Bourdieu 1984; Swartz 1997; Gorski 2013).^2^

Like Bourdieu's approach to classification struggles, worldview analysis in the sense advocated here can shed light on how worldviews become espoused, defended, and disseminated in a relational context: how they can mobilize followers and what their political implications are. Such approach does not reduce worldviews to an instrument in the hands of leaders but nevertheless looks at the effects they produce. The dynamic study of worldviews entails examination of how powerful actors or social groups enter into conflict and make themselves and their opponents objects of classificatory practices.

## Conflict Resolution and Worldviews

Conflict analysis can be approached in a variety of ways. Most conflict analysis has been preoccupied with the *causes* of conflict. Central themes have been incompatibility, unpredictability, fear, security dilemmas, and scarce resources.

During the Cold War conflict analysis was dominated by game theory, which was a tool to analyze the dynamics of great power conflicts. According to game theory, a conflict generates “rules” that determine the actions of the conflicting parties and comes to have a life of its own. Johan Galtung (1985) has been among the main exponents of this view, which understands conflict as a dynamic phenomenon where one party is reacting to the actions of the other, leading to further action and escalation of the conflict. Different concepts express these dynamics. In the field of international relations, Herz's notion of the “security dilemma” (Herz 1951) encapsulates the situation in which one state acts in fear of what the other state might do, and thus the preventive actions of one party feed into a pattern of escalating dynamics, where the other does exactly the same. Polarization and mirror imaging are other terms describing such dynamics, which create two irreconcilable camps and preclude options for acting differently and peacefully. The dynamic view of conflicts is applicable to a variety of players and contexts, perhaps because the actors are seen to be less important than the dynamics itself.

Although the major theories of conflict analysis, including the Galtungian approach, recognize that incompatibility is hard to eliminate to the utmost satisfaction of all parties, such theories nevertheless are based on the assumption that peace and order are functions of compatibility and agreement. This can be a reasonable assumption when dealing with classic conflicts over territory or other material interests in which incompatible demands for a scarce resource can hardly lead to a solution where both parties get what they claim is theirs. But in a conflict pattern related to differences in worldviews, one can question whether compatibility is the desirable ideal.

Developing a framework that fuses worldview analysis and the insights into the dynamics reflected in the security dilemma opens up a new path of thinking in conflict management and conflict resolution by scrutinizing the escalatory power of worldviews and investigating conflict dynamics that are not solely related to material interests. In line with what Herbert C. Kelman called an *interactive problem‐solving approach* (Kelman 2010), worldview analysis can bring to the fore the cognitive and emotional factors that shape a conflict. This approach to conflict resolution, which is anchored in a social psychological tradition, offers a lens for studying conflicts that is different than those focusing on the underlying structural causes of conflict or its strategic factors (e.g., realist or neo‐realist approaches). A social psychological approach to conflict analysis encourages researchers and mediators to attend to the worldviews of violent actors and to look for those recurrent discursive elements that appear in narratives of those engaged in conflict. The basic idea is that a conflict is a process driven by the “perception of collective needs, fears, [and] grievances” rather than the outcome of rational calculation (Kelman 2010: 194), and that the interpretation of events and acts plays a central role in the escalation and ending of any conflict. A war is not just a dispute about conflicting interests or territory or resources; a vital part of the conflict is always a definitional struggle in which the parties compete to assert their own truths as to what the war is about in the first place. Questions relating to the very nature of the war—who the aggressor is, the war's origins and context, and who is responsible for events leading up to it—can potentially have lasting impacts on the development of the conflict.

Kelman argued that while all conflicts might be embedded in incompatible goals and interests, territory, and resources, these discrepancies almost always echo and boost normative concerns about security and identity (Kelman 2007). To de‐escalate conflicts, identifying and describing the subjective perspectives of the parties is, therefore, crucial. Adding to this, the *intersubjective* dynamics between the conflicting parts is also important to understand.

Actions that acknowledge peoples' narratives can have a powerful psychological impact and open up a way to negotiation or other softening measures. As Kelman noted, expressing one's narratives can “regenerate people's sense of dignity and identity, allowing them to even let go of the conflict, even when it would not entirely remove the injustice they feel” (Kelman 2007: 205–206). Conversely, when fundamental grievances and beliefs are not acknowledged, the lack of acknowledgment can create a resistance to negotiate, even when both parties want to end the conflict and it is ripe for resolution. Notwithstanding the ripeness of the conflict, the fear of reaching an outcome through a negotiation process that may involve compromising a party's very existence and dignity can make it reluctant to extricate itself from violent actions (Kelman 2007). People's interpretation of events and of their position in the world can therefore supersede their rational calculus of the costs and benefits of engaging or disengaging from conflict. Worldview analysis brings attention to the proposition that incompatible interests and goals are not sufficient to keep conflicts alive. For conflicts to flourish and persist there must be stories of past grievances, traumas, sufferings, and redemptions that exacerbate the level of conflict by connecting it to questions of identity, self‐fulfillment, and justice. Acknowledging people's beliefs and values enhances their feelings of security and sense of identity, making compromise more likely.

## Conclusion: Collective, Emotional, Universal, but also a Particular Form of Conflict?

Worldviews are a collective and social phenomenon—shared perceptions of the world that are sometimes vividly expressed in world conflicts. Although there may be overlaps between worldviews and what some scholars might call ideology, culture, civilization, identity, state of mind, values system, or even zeitgeist, as this special issue of *Negotiation Journal* shows, conflict analysis would be enriched by bringing worldview analysis into the field. Indeed, some scholars in the field are taking more seriously the opposing visions of the world that sometimes seem to be at the center of conflict. They are not only taking them seriously as perspectives that matter, but they are avoiding classifying worldviews as empty rhetoric, as a cover for parties' real motivations, or as reflections of “mere extremism.” Some work already has been done that points to the polarizing mechanisms of certain worldviews (e.g., those that imply cosmic war images) but more research is needed on why some worldviews are more conflictual than others.

As described in this article, worldviews are shared. They are a way of making sense and order in the world—an explanation for why things are as they are—and they often offer justifications and rationales for how to “fix” things if there is disorder, conflicts, or violations of laws, behavioral norms, or moral or legal codes. Moreover, worldviews are not only intellectual sensemaking mechanisms. They also encompass ethical conceptions of good and bad as well as historical memories about greatness or injustices, and thus are able to mobilize collective emotions. This emotional dimension of worldviews and their mechanisms of mobilization is an area that requires further research.

To move the field forward and avoid falling into the trap of essentialism and the understanding of conflicts as static and immovable, several steps must be taken. Firstly, there ought to be space for examining the possibility that there are ways that individuals can navigate different worldviews at the same time or can transition from one to the other. What exactly are these mechanisms and when can worldviews coexist in the mind of an individual? Secondly, the conflict field needs to move from viewing worldviews as something that is relevant only for analyzing those at the margins of society, to viewing them as a universal condition and relevant for understanding all parties in a conflict. This can challenge the neutral ground some conflict parties often claim when describing the nature of a conflict. Naturally, there are substantive differences between the worldviews of a religious zealot from Texas, a Russian imperialist who wants to restore a perceived lost greatness, and a Danish defender of liberal democracies. However, function‐wise, a worldview can have similar effects in terms of mobilization, polarization, and evilization. Thirdly, the role of worldviews and how central they are to a conflict is variable. In one way they can be conceptualized as a dimension or part of every conflict. In another sense, worldviews can be viewed as a particular form of conflict requiring special attention as a conflict category of its own. In earlier work, I have described the particularities of doctrinal wars (Sheikh 2014) and the dynamics of “bundled” conflicts (Sheikh 2022). In both doctrinal wars and bundled conflict‐constellations, worldviews can be seen to play an escalatory role when such conflicts are centered on large‐scale principles, values, or doctrines. Where worldviews take a central role in a conflict and have an escalatory function, they can absorb other conflicts into a meta‐framework organized by the worldview's underlying assumptions. This complication of conflicts happened, for example, after the colonial era in the Middle East with the rise of Islamist movements across borders that reacted against repressive secularism. It also happened during the Cold War, with its divisive effects on the world; during the Bush‐led War on Terror in 2001 that was justified as a “defense” of freedom; and more recently, when Russia invaded Ukraine in 2022 and the conflict intensified by its portrayal as a battle between democracies on one side and autocracies on the other. A research agenda on worldviews and conflict analysis must include ways to advance our understanding of the differences between conflicts where worldviews play a role and conflicts that in their core are defined by differences in worldviews. Whereas the above examples reflect conflicts with worldviews at their center, local conflicts may also have worldview dimensions even though they are not yet part of a bundled conflict‐constellation that lifts the conflict to another scale. The local conflict‐lines in Afghanistan before the Soviets went into the country is an example of existing fault lines between communists and Islamists that still did not have worldviews at their center—access to power and privilege, and schisms between the urban and feudal appeared to be at least just as significant as differences in worldviews. This changed with foreign intervention, but also in tandem with how local factions interpreted their worldview differences. This sort of transformation—from worldviews being a part of the conflict to worldviews taking center stage—is a final point that deserves attention from the scholarly community.

## NOTES

^1^
This article draws on some of my earlier work on sociotheology and worldview analysis (Juergensmeyer and Sheikh 2013; Sheikh 2015, 2018, 2019, 2021).

^2^
According to Bourdieu, specialists have the authority to speak for a social collectivity—to articulate its history, political opinions, needs, and demands, and they therefore play a vital role in the maintenance of a group's boundaries and the mobilization of its members: “The ‘working class’ exists in and through the body of representatives who give it an audible voice and a visible presence” (Bourdieu 1991: 173).
